# Toward modular biological models: defining analog modules based on referent physiological mechanisms

**DOI:** 10.1186/s12918-014-0095-1

**Published:** 2014-08-16

**Authors:** Brenden K Petersen, Glen EP Ropella, C Anthony Hunt

**Affiliations:** 1 UCSF/UCB Joint Graduate Group in Bioengineering, University of California, Berkeley, CA, USA; 2 Tempus Dictum, Inc, Portland, OR, USA; 3 Department of Bioengineering and Therapeutic Sciences, University of California, San Francisco, CA, USA

**Keywords:** Modularity, Model reuse, Modeling & simulation, Mechanism, Agent-based model

## Abstract

**Background:**

Currently, most biomedical models exist in isolation. It is often difficult to reuse or integrate models or their components, in part because they are not modular. Modular components allow the modeler to think more deeply about the role of the model and to more completely address a modeling project’s requirements. In particular, modularity facilitates component reuse and model integration for models with different use cases, including the ability to exchange modules during or between simulations. The heterogeneous nature of biology and vast range of wet-lab experimental platforms call for modular models designed to satisfy a variety of use cases. We argue that software analogs of biological mechanisms are reasonable candidates for modularization. Biomimetic software mechanisms comprised of physiomimetic mechanism modules offer benefits that are unique or especially important to multi-scale, biomedical modeling and simulation.

**Results:**

We present a general, scientific method of modularizing mechanisms into reusable software components that we call physiomimetic mechanism modules (PMMs). PMMs utilize parametric containers that partition and expose state information into physiologically meaningful groupings. To demonstrate, we modularize four pharmacodynamic response mechanisms adapted from an in silico liver (ISL). We verified the modularization process by showing that drug clearance results from in silico experiments are identical before and after modularization. The modularized ISL achieves validation targets drawn from propranolol outflow profile data. In addition, an in silico hepatocyte culture (ISHC) is created. The ISHC uses the same PMMs and required no refactoring. The ISHC achieves validation targets drawn from propranolol intrinsic clearance data exhibiting considerable between-lab variability. The data used as validation targets for PMMs originate from both in vitro to in vivo experiments exhibiting large fold differences in time scale.

**Conclusions:**

This report demonstrates the feasibility of PMMs and their usefulness across multiple model use cases. The pharmacodynamic response module developed here is robust to changes in model context and flexible in its ability to achieve validation targets in the face of considerable experimental uncertainty. Adopting the modularization methods presented here is expected to facilitate model reuse and integration, thereby accelerating the pace of biomedical research.

## Background

Biomedical science can be characterized as the pursuit of deeper, more useful mechanistic insight into biological phenomena to facilitate advancing health. In silico modeling and simulation (M&S) can accelerate this process when models are designed for particular use cases. We define use case as a detailed, very particular description of the way a model will be used, including specifications for input, output, parameters, execution protocols, the interpretation of data, and qualitative and quantitative comparisons to other models. We follow software engineering terminology in referring to similarities between use cases as *usage patterns*. For a scientifically useful model, use cases must be highly specific and explicit, which enables clear specification of requirements. Such models are perpetual works in progress, satisfying various use cases throughout model development. In silico M&S methods are also viewed broadly as a promising countermeasure to the recent decline in pharmaceutical R&D productivity [1].

What M&S requirements must be satisfied to advance biomedical science and help reverse this productivity decline? There are many, including modularity, semi-autonomy of model components, and semi-automation of in silico experimentation and parameter searching. Further, biological software analogs must exhibit increasingly explanatory mechanisms and become increasingly biomimetic during execution. This work focuses on one requirement: modularity. Modularity is an important requirement in itself and also facilitates the satisfaction of other requirements. Namely, modularity is required to ease the reuse and integration of software analogs for the many particular, diverse use cases of individualized medicine and virtualized patients.

The heterogeneous nature of the objects of biomedical research and vast range of wet-lab experimental platforms make modularity especially desirable yet challenging. Biology is modular in its own sense, in that many phenomena are functionally separable [2]. Efforts to identify functional modules in biology entail identifying common causal event cascades or reaction networks [3]. On the other hand, biological components (e.g. cells) are not standardized; even individual cells within the same cell population can vary in phenotype [4]. Furthermore, wet-lab measurements in biology are complicated by the fact that many attributes are experimentally inaccessible, necessitating vastly different experimental platforms (e.g. in vivo, in vitro). The nature of biology and the challenge presented call for modular biological models designed to satisfy a variety of use cases.

But currently, most biomedical models exist in isolation. It is difficult to reuse or integrate models or their components for many reasons. An important reason is the lack of modularity or, if modular, a modularity method that fails to facilitate reuse and integration. Even in object-oriented models, in which model components are encapsulated into discrete software objects, components are usually tightly coupled yet not cohesive, rendering them difficult to be reused. Tightly coupled, non-cohesive models are those in which distantly related model components are highly interdependent. That is, they heavily rely upon each other via references, shared memory, or other a priori knowledge. Components of these models cannot be used in another model context without significant code refactoring. Modularization strives to circumvent these challenges, resulting in loosely coupled components that can function both in isolation and within the broader model context.

Modularity has established methods and demonstrated importance to M&S in many domains outside biology, including ecological modeling [5], agricultural modeling [6], and virtual manufacturing [7]. Established modularity methods also exist for particular modeling formalisms like discrete event systems specification (DEVS) [8],[9]. The term modularity is closely related to terms like model reuse [10], integration [6], and composability [11]; we focus here on reuse. Michael Pidd defines a *reuse spectrum* of modularity methods, within which we set the context for our modularization methods [10],[12]. At the right end of the spectrum is *code scavenging*, followed by *function reuse*, *component reuse*, and ultimately *full model reuse* at the left end. He notes that complexity of the required modularization methods increases from right to left, but the frequency in which model reuse is employed increases from left to right. Under Pidd’s spectrum, our modularization methods below focus on component reuse. Modular components (modules) allow the modeler to think more deeply about the role of the model and to more completely address a modeling project’s requirements. In particular, modularity facilitates component reuse and model integration for models with different use cases, including the ability to exchange modules during or between simulations. Having modules that have achieved degrees of validation separately and can be easily altered, both alone and when composed, will increase the pace toward better mechanistic explanations.

What is an appropriate target for component modularization in biological models? At specified levels of abstraction, many biological mechanisms are common to a variety of cell and tissue types. Paraphrasing Darden, we refer to mechanisms as entities and activities orchestrated such that they generate the changes characterizing a phenomenon from initiation to termination [13]. It is often at the mechanism level in which changes occur between normal and diseased states. Thus, software analogs of biological mechanisms are reasonable candidates for modularization. As an example, hepatocytes often respond to xenobiotic (e.g. drug) exposure by up-regulating (or down-regulating) xenobiotic metabolizing enzymes—a process called enzyme induction (or elimination) [14]. The cytochrome P450 family of enzymes metabolize xenobiotics according to specific metabolic pathways [15]. The intracellular event types are common to many pharmacodynamic responses [14],[15]. Thus, a generic pharmacodynamic response module that can be easily reused is expected to be useful within a broad range of biomedical models. Such physiomimetic mechanism modules (PMMs) are expected to broadly improve model usefulness without requiring significant refactoring of implementations.

### Existing efforts toward modularity

The need for modularity in biomedical M&S is broadly recognized, but existing efforts toward modularity are largely limited to equation-based modeling in the context of biochemical reaction networks. There are tools and frameworks for developing modular equation-based models of biochemical reaction networks, including metabolic, signaling, and regulatory pathways [16]–[20]. Typically, these modularization approaches involve breaking down a larger hypothesized network into smaller components or “modules” that are then recomposed to form a coherent whole [16]. The unit of modularization is a set of equations that usually maps to one part of a hypothesized biochemical reaction network. We refer to these modularity methods collectively as “network-based” modularity. The following examples illustrate the state-of-the-art. Note that, while most of the examples below are developed within the context of biochemical reaction networks, some of them can be applied more broadly.

 Mallavarapu et al. provide a framework for developing and reusing equation-based models, confined to ordinary differential equation models following the chemical master equation [17]. The framework facilitates abstraction, which allows generic properties of components or sub-systems to be specified independently of specific instances. Their methods also focus on hiding internal complexity of each module and allowing user-defined rate functions.

 ProMot is a tool that facilitates modular setup and editing of network models, including biochemical reaction networks [18]. It is unique in its support of both differential algebraic equation systems and Boolean network models.

 In the context of synthetic biology, TinkerCell allows for the modular construction of ordinary differential equation models describing genetic circuits or regulatory networks [19]. A user creates models from a list of “parts,” and the program assigns rate equations describing dynamics of transcription and translation reactions. The unit of modularization is an equation that maps to a “biological part,” defined as “a functional unit of DNA that encodes for a specific biological function” (http://parts.igem.org/Help:Parts).

 Snoep et al. demonstrate integration of many sub-network modules, aimed at modeling the entirety of biochemical processes in a living cell [20].

 Blinov et al. semi-automate modularization by first extracting sub-systems (modules) of biochemical reaction networks and then specifying the rules of module interaction [16].

While network-based modularity may be an essential requirement for its specific model type(s) and use cases within its specific domain, accelerated generation of increasingly explanatory mechanisms requires more generalized modularization methods. A future research question, which is beyond the scope of our current work, is when and how network-based modularity methods might be employed productively within more generalized modularization methods. The efforts described above have limitations. Namely, they are inextensible to model types other than equation-based models. Each method above is limited to one or a few model types—usually a combination of ordinary differential equations (deterministic or stochastic), differential algebraic equations, and Boolean logic. Many of the supported equations are further limited to a specific functional form, e.g. ordinary differential equations following the chemical master equation [17]. These methods are inextensible to other model types, which is significant given that other model types, including agent-based, actor-oriented, discrete-event, and hybrid, are proving increasingly useful [1],[21]. Further, most of the methods above rely on using standardized markup languages and/or ontologies. Models or modules that cannot be described using supported markup languages and/or ontologies cannot be modularized or integrated using the above methods, absent considerable reengineering. As examples, the modularity methods of Blinov et al. require interchangeable modules to be described using either the Systems Biology Markup Language (SBML) or their proprietary markup language [16]; TinkerCell uses strict ontologies for model components like parts, promoters, and transcription factors, without the capability of editing or defining new ontologies [19]. The exception is Mallavarapu et al., whose platform circumvents this limitation by allowing user-defined ontologies. They note that ontologies, like those available in SBML, are agreed upon by the community and cannot be altered by any one user [17]. The greater flexibility of user-definable ontologies is essential when a goal is improved mechanistic explanatory power. Often, non-equation-based models cannot be feasibly described using standard markup languages and/or ontologies, especially when those models are upstream in the development process. Thus, modularity methods must be applicable even to those models that are difficult or not possible to describe using those languages.

From the perspective of multi-scale, multi-attribute modeling, existing methods are not without additional practical problems. Even when restricted to the appropriate model type and domain context, current reuse and integration efforts often require significant model-specific refactoring. For example, Palsson et al. integrate three distinct models of immune cell interaction using a modular framework; included is a three-page table summarizing the model-specific integration issues they overcame [22]. Thus, accelerated progress requires more generalized modularization and integration methods that facilitate limiting, if not eliminating, model-specific refactoring.

The limitations above inform us of the challenges to address in a more generalized modularization method. Namely, the methods should not be limited to a single model type (i.e. equation-based models), domain context (i.e. biochemical reaction networks), or markup language (e.g. SBML). Drawing on many of the advantages from the above examples, herein we favor a “physiologically-based” modularization method (as distinct from “network-based” modularity) aimed at overcoming these challenges. While the methods here are extensible to strictly equation-based models, our drive to achieve new, demanding requirements [1] forces us to focus on other model types (including hybrid models that include equation-based models) that do not yet have established modularization methods.

It is worth noting that methods will or should always be driven by requirements, whether or not the requirements are explicitly stated. For most existing methods, the modularity goal is to reuse or curate biochemical reaction network models (whole models or sub-models) expressed as systems of mathematical equations. Achieving this goal entails the following requirements:

 Methods are applicable to a subset of equation types.

 Methods can be described using standardized markup languages and/or ontologies.

 Methods are independent of modeling framework or programming language.

The generalized modularity goal is different. It is to reuse, repurpose, or integrate model mechanisms within different model use cases of potentially different model types. Achieving this goal entails the following requirements:

 Methods are applicable to a variety of model types and methods, beyond equation-based models.

 Methods need not be restricted to models describable using standardized markup languages and/or ontologies. Allow user-defined (or redefined) ontologies that may be needed as mechanistic insight improves.

 Methods are independent of modeling framework or programming language.

A final example of a modularity approach, with requirements more closely aligned to the generalized modularity goal, is exemplified by Liu et al. [23]. They employ an object-oriented strategy to integrate four models of angiogenesis in skeletal muscle, focused on exchanging data among modules written in different programming languages. A comparison to our approach is given in Methods.

### Our contributions

In this work, we present a generalized method of modularizing mechanisms into software objects that we call PMMs. We then demonstrate the approach by applying these methods to develop a generic pharmacodynamic response module. Since most existing models were not initially constructed with modularity in mind [17],[22], we developed the methods to be either applied to existing, non-modular models or applied at the start of model development. PMMs are designed to be reusable, biomimetic analogs reflecting current understanding of the underlying biology. They are designed to be flexible in their ability to produce results that are indistinguishable from wet-lab experiments embedded in considerable uncertainty. Their design enables mechanisms to be as robust as feasible to changes in model context, including model-to-time mappings. We exemplify these capabilities by achieving validation targets from two different experimental platforms. To do so, we modularize four intracellular mechanisms adapted from an in silico liver (ISL). The four analog mechanisms map to enzyme induction, enzyme elimination, drug metabolism, and enzyme-substrate binding. We first verify that the modularization process itself did not alter simulation results. The modularized ISL then achieves validation targets drawn from in vivo propranolol outflow profile data.

In addition, we create a modularized in silico hepatocyte culture (ISHC-modular, hereafter referred to as ISHC). It uses the same PMMs as the ISL and required no refactoring. Though the PMMs are identical, groundings are relational and thus free to differ between the ISL and ISHC, and we demonstrate the value of enabling that freedom. We describe ISHC validation against in vitro propranolol intrinsic clearance data from multiple sources. The feasibility of this task has been demonstrated in an earlier model in Sheikh-Bahaei and Hunt [24], and we present several extensions of that model. In this work, the ISHC demonstrates exchangeability and reusability of PMMs and the ability to separately validate software mechanisms against validation data spanning multiple wet-lab platforms.

### Model use cases

The ISL and ISHC are biomimetic analogs. A biomimetic analog is a software model that, when executed, produces phenomena that mimic attributes measured or observed during referent wet-lab experiments [1]. Unlike traditional inductive models, biomimetic analogs also rely on abductive [25] and analogical [26] reasoning. Most mathematical (e.g. equation-based) models are inductive: they start with data and then infer computational mechanisms that might produce that data; for example, fitting an equation to a pattern observed in data [21]. In contrast, analogs rely on abductive reasoning: they start by focusing on a phenomena of interest, hypothesizing model mechanisms, synthesizing an explanatory composition of those mechanisms, and then testing that composite model against data. Whereas inductive models are useful for positing explanations of patterns observed in large amounts of data and when precise prediction is required, abductive models are useful for exploring mechanistic hypotheses and when uncertainty is pervasive [21],[27]. Thus, analogs are suitable for experimentation and mechanistic hypothesis testing. An experiment on an analog is called an in silico experiment. It is precisely analogous to a wet-lab experiment.

The ISL simulates drug clearance experiments in an in vivo setting. One use case configuration maps to a portion of an in situ isolated, perfused rat liver. An important use case is the multi-indicator dilution technique to measure the hepatic outflow profile in response to a bolus of drug [28]–[32]. At the start of this experiment, the bolus is administered to the portal vein. As blood flow carries drug through the sinusoid tract to the central vein, a fraction of drug is taken up by hepatocytes, where it may be metabolized. Central venous outflow is collected at distinct time intervals and the amount of drug in each elution is measured, resulting in an outflow profile curve. Because a fraction of drug reaches the central vein without being metabolized or taken up by hepatocytes, the outflow profile yields a characteristic peak anywhere from a few seconds to several minutes after the initial drug administration [32]. Features of the outflow profile curve depend on factors including liver disease state [29], drug lipophilicity [30],[32], and sinusoid volume [31].

The ISHC simulates drug clearance experiments in an in vitro setting. It maps to a portion of a monolayer culture of isolated rat hepatocytes. Its use case is measuring hepatocytes’ intrinsic clearance—a measure of the intrinsic ability for hepatic enzymes to metabolize drug [33],[34]. At the start of this experiment, a bolus of drug is administered into the surrounding media, and hepatic enzymes metabolize drug as it is exchanged between cell and media. The amount of remaining drug is measured over time, and the resulting curve is used to calculate the hepatocytes’ intrinsic clearance. Intrinsic clearance is often used as a predictor of various in vivo clearance measures, including hepatic clearance [35], in vivo intrinsic clearance [34],[35], and extraction ratio [36].

## Methods

To avoid ambiguity between in silico components and their referent biological counterpart, capitalization is used when referring to the former, e.g. Hepatocyte. PMMs are named using the suffix Handler, e.g. MetabolismHandler. Java Interfaces are named using the suffix Info, e.g. CellInfo. Parameter names are italicized. For a full list of ISL and ISHC parameters and their explanations, see Table 1.

**Table 1:** ISHC parameters details and descriptions, including whether that parameter also exists in the ISL

**Parameter name**	**Type/Range**	**Example**	**In ISL?**	**Description**
**Simulation control parameters**				
*seed*	natural	12345	Yes	Random number generator seed.
*cycleLimit*	natural	120	Yes	Cycles after which to stop the simulation.
*monteCarloTrials*	natural	16	Yes	Number of Monte Carlo trials to execute.
**Whole model parameters**				
*dosage*	natural	10000	No	Number of Solutes to administer at the start of the simulation.
*pExitMedia*	[0.0,1.0]	0.15	No	Random draw from *U*(0,1) ≤ this value determines whether a Solute can move from MediaSpace to CellSpace.
*pExitCell*	[0.0,1.0]	1.0	No	Random draw from *U*(0,1) ≤ this value determines whether a Solute can move from CellSpace to MediaSpace.
*scale*	integer	50	No	Number of Solutes that can fit in one grid point in CellSpace.
*hepDensity*	[0.0,1.0]	0.9	Yes	Fraction of grid points in CellSpace that contain a Hepatocyte.
**Hepatocyte parameters**				
*pBind*	[0.0,1.0]	0.1	Yes	Random draw from *U*(0,1) ≤ this value causes a Binder to bind a Solute.
*bindersPerCellMin*	integer	4	Yes	Minimum for a uniform random draw setting initial number of Binders in a particular Cell.
*bindersPerCellMax*	natural	8	Yes	Maximum for a uniform random draw setting initial number of Binders in a particular Cell.
*bindCycles*	natural	2	Yes	Number of simulation cycles a Solute stays bound to a Binder.
**Solute-specific parameters**				
*membraneCrossing*	boolean	TRUE	Yes	Indicates whether this Solute type can partition into Cells.
*pMetabolize*	[0.0,1.0]	0.1	Yes	Random draw from *U*(0,1) ≤ this value causes an Enzyme to metabolize the Solute to which it is bound.
*tag*	string	Metabolite	Yes	Name for this type of Solute (e.g. Drug, Metabolite).
*ratio*	[0.0,1.0]	0.5	Yes	Fraction of this type of Solute to create.
**Induction/elimination parameters**				
*eiThresh*	natural	1	Yes	Threshold above which the induction accumulator triggers an induction event.
*eiRate*	≥ 0.0	0.05	Yes	Rate at which Enzymes can be created.
*eiResponse*	≥ 0.0	0.25	Yes	Number of Enzymes to induce when an induction event is triggered.
*elThresh*	natural	1	Yes	Threshold above which the elimination accumulator triggers an elimination event.
*elRate*	≥ 0.0	0.05	Yes	Rate at which Enzymes can be destroyed.
*elResponse*	≥ 0.0	0.25	Yes	Number of Enzymes to eliminate when an elimination response is triggered.

In the ISL and ISHC, Solutes are mobile objects that map to a group of small molecules. Solutes can have any number of properties that map to pharmacodynamic properties. Each Solute is assigned a type: one of various Drugs or Metabolites. Binders are objects that bind (associate with) Solutes. Enzymes are a subtype of Binders that can metabolize bound Solutes. Solutes can either exist inside or outside Cells; herein Binders exist only within Cells. Cells are objects that map to groups of cells, and can be further delineated into Hepatocytes and Epithelial Cells. All Cells can undergo binding between Solute and Binder; however, metabolism, induction, and elimination events occur exclusively in Hepatocytes. Analog time advances in simulation cycles. The above capitalized entities establish an implicit ontology, inherently semantic with respect to the model and components.

We describe two sets of modularization methods. The first is general and scientific, applicable to any multi-scale biological model; the methods can be applied using almost any programming language and modeling environment. The second is specific and engineering-focused; it describes how we specifically applied the general methods to the ISL and ISHC.

### General modularization methods

**Figure 1:**
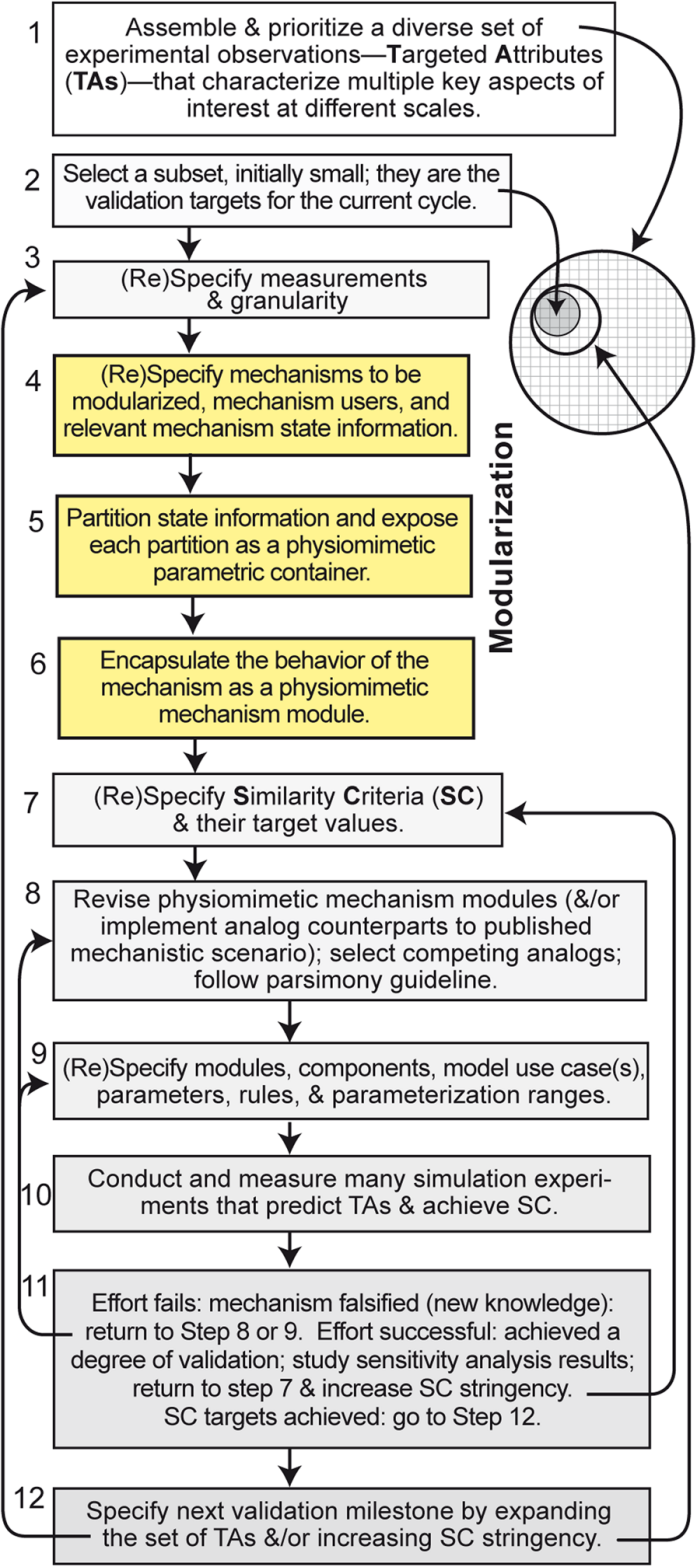
**An iterative protocol for refining and modularizing biomimetic analogs.** Steps 4 – 6 encompass the general modularization methods.

The following list describes the general modularization methods. We set these methods within a broader biological M&S approach called the iterative refinement protocol (IR Protocol) [27]. The IR Protocol provides a scientific method for developing and validating multi-scale biological models that are increasingly biomimetic. It focuses on iterative model refinement following a strict parsimony guideline. In Figure 1, we present the methods below as an extension of the IR Protocol.

(1) **Mechanisms** – Identify the software mechanisms to be modularized. These mechanisms may be algorithms, rules or governing logic, sets of equations, or other computations. Mechanisms need not map to specific biological or physiological functions, but should do so if they are to maximize the benefits of modularization listed in Discussion. Examples of common mechanism types in biology include: movement and transport (e.g. cell movement; diffusion; endocytosis; ligand-receptor trafficking); biochemical reaction networks (i.e. metabolic, signaling, and regulatory pathways); biophysical phenomena (e.g. force transduction; action potential firing); changing activity state (e.g. T cell activation in response to antigen presentation); damage, injury, and repair (e.g. in response to reactive oxygen species); disease progression; therapeutic intervention; cell growth, division, and morphogenesis; and induced environmental changes (e.g. cell culture incubation, shaking).

(2) **Users** – Identify the objects intended to use the PMMs. A mechanism user may be an entire model or a component. Again, each mechanism user need not map to a specific biological or physiological entity, but should do so if it is to maximize the benefits of modularization listed in Discussion.

(3) **State information** – Identify the state information used and/or altered by the mechanisms; that is, all parameters and input/output variables, including any units associated with these quantities. State information can be of any data type, specific or not to the modeler’s framework.

(4) **Partition** – Partition the state information into physiologically meaningful or similar groupings. For example, variables mapping to concentrations of different mobile objects may be partitioned into one group, and variables mapping to rate constants in another. Partitioning decisions will be influenced by many factors, including model use case and mechanism granularity.

(5) **Exposure** – Create parametric containers, named according to the physiological information they contain. Parametric containers must be able to find and expose individual sub-elements within the state information at runtime. So doing allows the mechanism user to ask for specific data deemed useful while ignoring unknown or unused information. The mechanism user provides the correct data types and/or units required by the parametric container; data type and/or unit conversion may be necessary. Placing this responsibility on the mechanism user allows new or different mechanism users to execute PMMs without altering or refactoring PMM code. The implementation will depend on the programming language and modeling environment. Examples include using: Java, C++, or .NET Interfaces; Objective-C protocols; Scala traits; XML schemas in XML-based languages;or JSON, for use in web-based applications.

(6) **Encapsulation** – Implement and encapsulate the behavior of each mechanism in the appropriate language of the environment. In the implementation, use the parametric containers to hold, access, and/or manipulate state information. The encapsulated mechanism is a PMM. Again, the implementation will depend on the programming language and modeling environment. Examples include using: Java, C++, .NET, Objective-C, or Scala Classes; threads or processes; Promises/Futures or Actors, for use in the actor model of computation; or XSLT or XPath, for use in XML-based languages.

To summarize, there are three key software aspects comprising the above mechanism modularization process: physiomimetic parametric containers, the PMM, and the mechanism user. Parametric containers facilitate communication of state information between PMM and mechanism user: they identify which state information is required by the mechanism user to use a particular PMM. The PMM executes the mechanism logic: it uses the parametric containers to access and manipulate the contained state information. The mechanism user is the entity using the PMM: it exposes the required state information as parametric containers and executes the PMM.

### ISL and ISHC modularization

**Figure 2:**
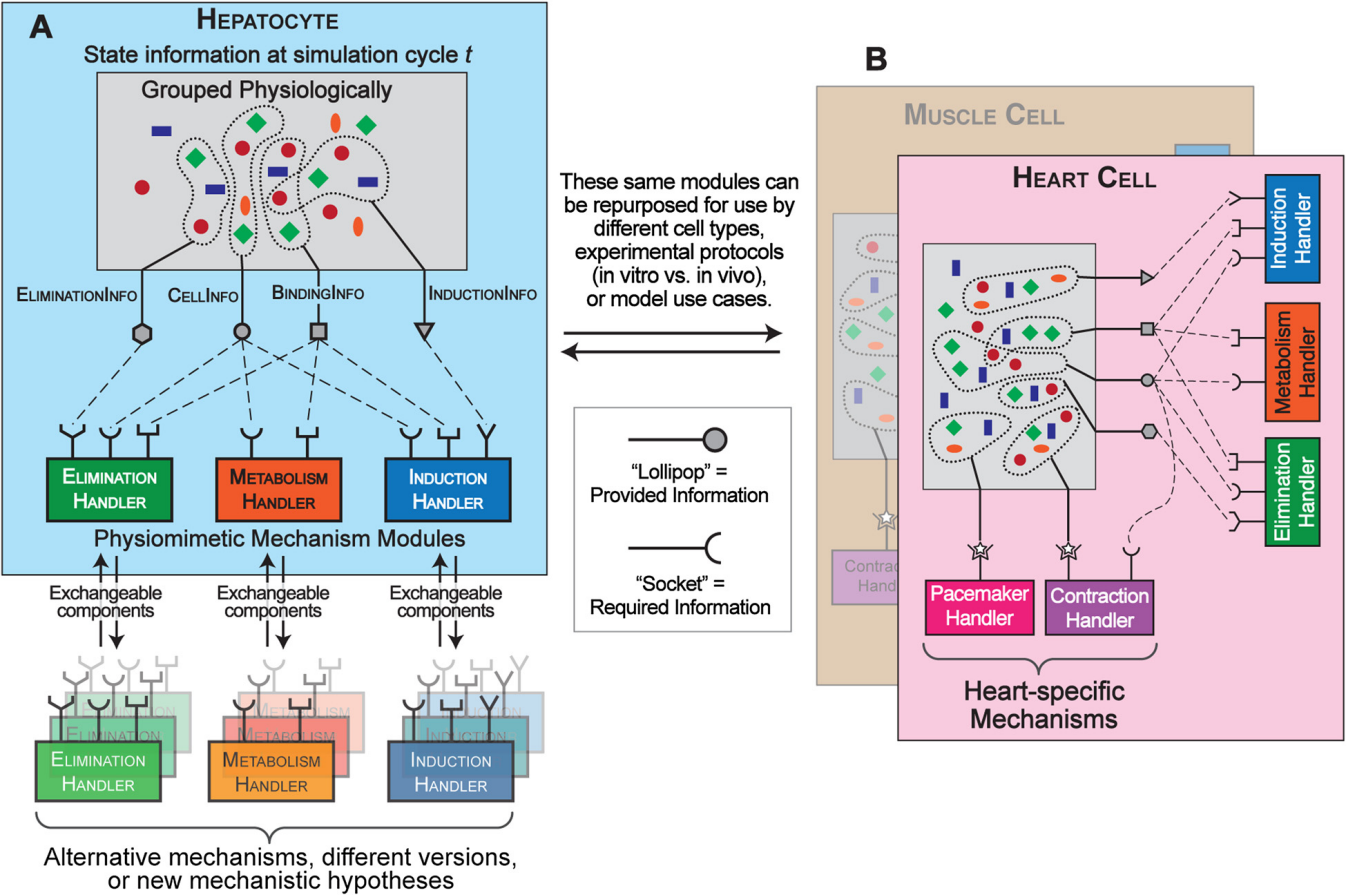
**Exchangeability and reusability of physiomimetic mechanism modules.** “Lollipops” represent parametric containers: state information exposed via Java Interfaces. “Sockets” represent state information required as parameters via Java Interfaces. **A)** Hepatocyte component diagram and exchangeability. Hepatocyte state information is grouped physiologically and exposed to PMMs as Java Interfaces. These PMMs are easily exchanged with alternative mechanisms, different versions, or new mechanistic hypotheses. **B)** Alternatively, these same PMMs can be used by different cell types, experimental protocols (in vitro vs. in vivo), or model use cases. The only requirement is that the mechanism user (e.g. Heart Cell) exposes the appropriate groups of state information as Java Interfaces. It can then include its own, heart-specific, modular or non-modular mechanisms without interfering with the PMMs.

The following list of method descriptions, illustrated in Figure 2, provides details for our application of the above methods to the ISL and ISHC. Furthermore, it generalizes our implementation to biological models written in Java. Steps 1 – 4 above are independent of modeling environment; thus, steps 1 – 4 below simply describe how we applied them to the ISL/ISHC. Steps 5 and 6 above depend on the modeling environment; thus, steps 5 and 6 below first describe our implementation generalized to models written in Java, and then describe how we applied this approach specifically to the ISL/ISHC. Though the details in steps 5 and 6 below are Java-specific, the implementation easily translates to other programming languages supporting software interfaces.

(1) **Mechanisms** – The mechanisms are four intracellular mechanisms of the ISL [37]: enzyme induction, enzyme elimination, metabolism, and binding. Prior to modularization, the logic governing enzyme induction, enzyme elimination, and metabolism were handled together within the Hepatocyte object. Binding events were handled within the Cell object, from which Hepatocyte derives. The remainder of this description focuses on the metabolism mechanism.

(2) **Users** – The users of the metabolism mechanism are Hepatocytes.

(3) **State information** – The state information used by the metabolism mechanism includes (A) drug objects, (B) enzyme objects, (C) bound drug-enzyme pairs, (D) metabolism probabilities, (E) metabolite production probabilities, and (F) the types of Solutes included in the simulation. The ISHC is grounded relationally (see Relational Grounding below), so analog-to-referent mappings to real-world units depends on use case and is handled post-simulation.

(4) **Partition** – We partitioned the state information into two groups: (A)-(C) above are related to drug and enzyme binding, whereas (D)-(F) are specifically related to metabolism.

(5) **Exposure** – *Generalized Java implementation*: we exposed each partition using Java Interfaces that contain methods to access and manipulate the state information. A Java Interface defines a behavior protocol to which unrelated classes of objects can adhere using potentially very different implementations. Here, each Java Interface contains methods to access or manipulate the physiological state information in its group. *Applied to the ISL and ISHC*: the first partition is exposed as a Java Interface called BindingInfo. It contains methods to access (A)-(C) above. The second partition is exposed as a Java Interface called MetabolismInfo. It contains methods to access (D)-(F) above. Since the addition or removal of Solute may be defined differently in different modeling frameworks, MetabolismInfo also includes methods to add or remove Solute.

(6) **Encapsulation** – *Generalized Java implementation*: for each PMM, we defined a new Java Class, each with a single function called “run” that takes Java Interfaces as parameters. We copied the pre-existing governing logic into the “run” function and replaced references to state information with corresponding methods from the Java Interfaces, thereby encapsulating the mechanism. Mechanism users then implement the Java Interfaces. To use a PMM, a mechanism user instantiates it and calls its “run” function when desired. *Applied to the ISL and ISHC*: we created the Java Class MetabolismHandler, whose “run” function takes BindingInfo and MetabolismInfo as parameters. Hepatocyte implements BindingInfo and MetabolismInfo as follows. Java Interface methods to access (A)-(F) above simply return the corresponding state information. MetabolismInfo methods to add (or remove) Solute do so by adding (or removing) a Solute object to (or from) three locations: a master list of all Solutes, a Hepatocyte-specific list of Solutes, and the grid space at the location of the Hepatocyte. To use MetabolismHandler, a Hepatocyte instantiates it and calls its “run” function once per simulation cycle.

We summarize the Java-specific modularization methods applied to the ISL and ISHC by describing the same three key software aspects. The physiomimetic parametric containers are Java Interfaces, e.g. BindingInfo and MetabolismInfo. They contain methods to access and manipulate state information. The PMMs are Java Classes, e.g. MetabolismHandler. They take Java Interfaces as parameters and use the contained information to execute the mechanism logic. The mechanism users are Hepatocyte and Cell. They implement the Java Interfaces and execute the PMM once per simulation cycle. Java Interface implementation may require unit conversion when the mechanism user provides data in different units than required by the PMM. Once each Java Interface is implemented, the mechanism user is free to use any PMMs requiring any combination of these Java Interfaces. Abridged Java code for representative examples of each of the three key software aspects is available in Additional file 1.

The following example demonstrates the three software aspects in the ISL and ISHC. CellInfo (the physiomimetic parametric container) contains an accessor for a variable that maps to the resources (e.g. O_2_) made available to the cell. InductionHandler (a PMM) uses this information to determine the capacity to up-regulate Enzymes. Hepatocytes (the mechanism users) implement the Java Interface differently in the ISL and ISHC. In the ISL, a Hepatocyte implements the resources accessor by applying a gradient function related to its distance from the portal vein, which maps to the decrease in available resources deeper into the tissue. In the ISHC, a Hepatocyte implements the accessor as a constant value because the monolayer of cells directly contacts well-mixed culture media. Thus, the two different models utilize the same PMMs but with different Java Interface implementations.

The parametric containers required by a particular PMM are based on available wet-lab experimental data related to the referent mechanism. Continuing the above example, enzyme induction has been found to be influenced by drug and enzyme identity and cell type [38]. To represent this knowledge, the PMM InductionHandler requires the Java Interfaces BindingInfo (containing lists of drugs and enzymes), CellInfo (containing information regarding cell type), and InductionInfo (containing additional parameters specific to enzyme induction). Thus, the mechanisms are based on referent physiology. However, we prefer the qualifier “physiomimetic” over “physiologically-based” to emphasize distinctions between model and referent that cannot be ignored. That emphasis (like using capitalization) helps avoid model reification. Reification is ignoring or failing to recognize this distinction; it is a logical fallacy in which model and referent are confused or conflated [39].

Since PMMs are packaged into distinct software entities, they can easily be exchanged for alternative mechanisms, different versions, or new mechanistic hypotheses within the same analog (Figure 2A). These different versions may exhibit different mechanistic granularities and/or represent different cell states (e.g. normal vs. diseased). PMMs can even be exchanged “on the fly”—at any time *within* a simulation. Note that such dynamic module replacement may incur technical challenges, and we point the reader to established guidelines and technical conditions to facilitate successful on the fly PMM exchange [40],[41].

By design, a particular PMM is not aware of the existence of its mechanism user; that is, it requires no a priori knowledge of the mechanism user. Rather, PMMs communicate with the mechanism user via parametric containers, which are not specific to the mechanism user. Thus, completely different models can reuse and/or repurpose PMMs simply by exposing the appropriate state information in parametric containers (Figure 2B). For example, rather than requiring Hepatocyte-specific parameters, PMMs developed here only require generic parameters like BindingInfo. Thus, a hypothetical Heart Cell can use the pharmacodynamic response PMMs originally used within Hepatocyte. Additional mechanisms used by Heart Cell—whether or not they have been modularized—will not be disrupted by the use of pharmacodynamic response PMMs.

The general method we outline and exemplify using Java Interfaces is also exemplified by the specifics of Liu et al. [23]. They encapsulate mechanisms (flow, regulation, and capillary formation) as modules, use shared memory for information exchange between modules, provide for dynamic loading and parameterization of interface implementation, and explicitly design inter-module mapping interfaces. Because their integration is driven in a fundamental way by the high computational expense of the mechanisms, it is a low-level integration, thereby requiring a very technology-oriented description. Although the methods used may seem different than those presented herein, there are similarities. An important distinction, however, is that, for this work’s use cases, our strict parsimony guideline allows us to operate largely within the high-level Java environment, far above the virtual machine, whereas computational requirements of Liu et al. demand that they puncture the virtual machine layer and implement their modularity at and below the interface between the virtual machine and the underlying language runtimes. To engineer a fully general software infrastructure facilitating either their or our methods would require “lifting” the model-specific modularizations into a standardized architecture for plugins. The general method we outline is intended to help guide that engineering effort.

### Logic governing the pharmacodynamic response PMMs

Induction and elimination mechanisms are detailed in Ropella et al. [42]. The mechanisms function by creating (induction) or removing (elimination) Enzymes as a function of the number of Drugs present in the entity using the mechanism (i.e. Hepatocyte). An important parameter is *resources* (see previous section). For induction, the number of Enzymes to be created varies inversely with *resources*. For elimination, the number of Enzymes to be removed varies directly with *resources*. This is consistent with the findings that oxygen-rich environments result in increased elimination and decreased induction of drug-metabolizing enzymes both in vivo [43],[44] and in vitro [45].

The metabolism mechanism samples each Solute in the map of bound Solute-Binder pairs. For each Solute, a pseudo-random (hereafter, random) draw from the uniform distribution, *U*(0,1), determines whether the Solute is metabolized. The probability of metabolism is based on a Solute-specific parameter, allowing different types of Solutes to have different metabolism probabilities. A metabolized Solute is replaced with a Metabolite. If the Solute can produce multiple types of Metabolites, one is randomly selected using a probability map. Otherwise, the metabolized Solute is simply removed from the system. This mechanism is kept sufficiently general so that additional users of MetabolismHandler can implement their own model-specific metabolic pathways by changing Solute-specific parameters rather than refactoring the entire mechanism.

The binding mechanism samples each unbound Solute inside a Cell. For each Solute, a random draw from *U*(0,1) determines whether the Solute binds (with probability *pBind*) to one unbound Binder in that Cell, forming a bound Solute-Binder pair. Upon binding, the Solute is scheduled to be released from the Binder after *bindCycles* simulation cycles. Scheduling events (like Solute release) depends on the specific modeling framework being used. Since we expect others using PMMs to utilize different modeling frameworks, the user of BindingHandler must implement scheduling Solute release as part of implementing the BindingInfo Java Interface.

### Model development

The ISL and ISHC are both implemented in Java, utilizing the MASON multi-agent simulation toolkit [46]. They are object-oriented, multi-scale, agent-based, discrete-event simulation systems. They are used to falsify (or achieve validation targets for) software mechanisms that serve as mechanistic hypotheses about, for example, acetaminophen-induced hepatotoxicity in an in vivo (ISL) or in vitro (ISHC) setting. Both analogs use the same PMMs but have different model structure and operating principles. As we discuss ISL and ISHC specifics, refer to Table 2 for important similarities and differences between them.

**Table 2:** Similarities and differences between ISL and ISHC

	**ISL**	**ISHC**
**Referent system**	In situ isolated, perfused rat liver	In vitro rat hepatocyte culture
**Targeted attribute**	Outflow profile	Intrinsic clearance
**Similarity criteria**	80% of points fall within band of ±1 standard deviation of wet-lab value	Value falls within ±1 standard deviation of wet-lab value; *r*^2^ > 0.95
**Structure**	Concentric, cylindrical grids within sinusoidal network	Stacked, two-dimensional, rectangular grid system
**Time scale**	~Seconds/minutes	~Minutes/hours
**Test drug**	Propranolol	Propranolol
**Physiomimetic mechanism modules used**	InductionHandler, EliminationHandler, MetabolismHandler, BindingHandler	InductionHandler, EliminationHandler
		MetabolismHandler, BindingHandler

### ISL development

**Figure 3:**
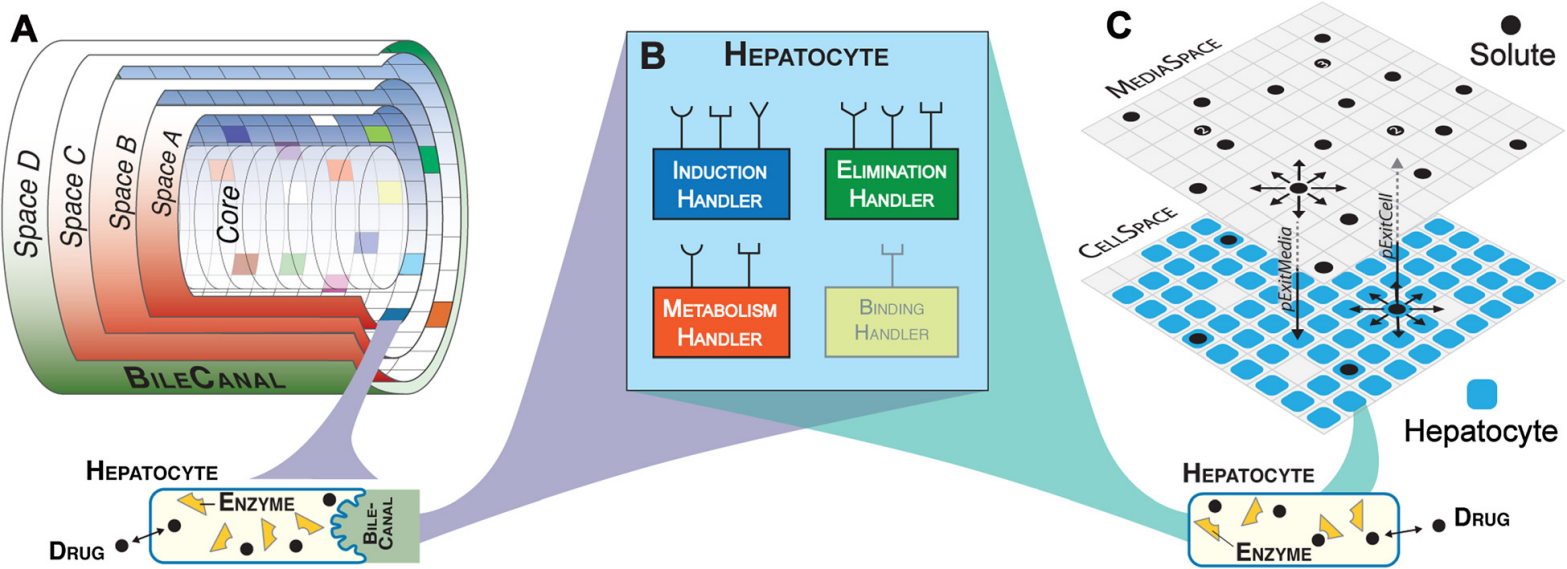
**Structures of the ISL and ISHC. A)** An ISL Sinusoid Segment. The Sinusoid Segment is the functional unit of the ISL. It contains thousands of Hepatocytes and Endothelial Cells contained within distinct spaces. **B)** Simplified component diagram of a Hepatocyte. Only PMMs are shown, highlighting the fact that the ISL and ISHC share the same PMMs. BindingHandler is grayed to emphasize that it belongs to the Cell class, from which Hepatocyte derives. **C)** ISHC structure. The ISHC contains two grids: CellSpace and MediaSpace (only portions of each grid are shown). Drugs can move laterally within a grid, or between CellSpace and MediaSpace, subject to the parameters *pExitMedia* and *pExitCell*.

Full details of the ISL structure and operating principles are provided elsewhere [37],[42],[47]–[50]. Briefly, the ISL consists of a directed graph—or sinusoid network—of interconnected nodes and edges. Each node is a Sinusoid Segment. Edges map to direction of blood flow, from the portal vein to the central vein. The Sinusoid Segment is the functional unit of the ISL. Each Sinusoid Segment contains hundreds of Cells contained within distinct spaces (Figure 3A). The innermost space is the Core, which maps to blood carrying referent compounds through sinusoid networks from portal vein to central vein. The outermost space is the Bile Canal, which contains Solutes excreted from Cells. Between the Core and Bile Canal are concentric cylindrical grids that can contain hundreds of Cells. Immediately surrounding the Core is an empty grid, followed by a grid containing Endothelial Cells; the next grid contains Hepatocytes. Each simulation cycle, the ISL reports the fraction of Drug objects contained in the Central Vein. The resulting plot is an ISL outflow profile (see Additional file 2).

### ISHC development

ISHC components and scales are illustrated in Figure 3B-C. The ISHC structure is composed of two stacked, rectangular grids, each mapping to different in vitro spaces. CellSpace maps to the monolayer of hepatocytes, and each grid point contains at most one Hepatocyte. MediaSpace maps to culture media. Both grids may contain Solutes. Solutes can move between grids or laterally within a grid. We recognize that there are many alternatives to using two-dimensional grids to map to referent spaces. For example, we could implement continuous space in which Hepatocytes have distinct area or volume. Alternatively, MediaSpace could be a three-dimensional grid in which height is explicit. We chose stacked, two-dimensional grids because doing so is both parsimonious and computationally inexpensive. However, when future use cases or the addition of new targeted attributes require alternative representations of space, the current ISHC structure will be falsified and iterative model refinement will lead to a new structure.

At the start of a simulation, Hepatocytes are randomly assigned to grid points on CellSpace. Each Hepatocyte contains Enzymes. The number of Enzymes in each Hepatocyte is subject to a uniform random draw between the parameters *bindersPerCellMin* and *bindersPerCellMax*. Drugs are then randomly assigned to MediaSpace grid points. Total Drugs equal the value of the parameter *dosage*. Hepatocytes are randomly selected for execution each simulation cycle. During execution, a Hepatocyte executes its four mechanisms modules—MetabolismHandler, BindingHandler, InductionHandler, and EliminationHandler—in random order. The analog’s state information, for example the number or locations of Solute objects, changes as a consequence of mechanism execution.

During each simulation cycle, each Drug has a chance to move one grid point: laterally (within the same space), downward (from MediaSpace to CellSpace), or upward (from CellSpace to MediaSpace). For a Drug to move into or out of a Hepatocyte, the Solute-specific Boolean parameter *membraneCrossing* must be true. A Drug attempting to move downward or upward is further subject to a random draw from *U*(0,1) using the parameters *pExitMedia* and *pExitCell*, respectively. These parameters map to an encapsulation of the ability for drugs to enter or exit hepatocytes via cell-surface transporters or simple diffusion [51]. We invoked the following constraints on parameterization choices to facilitate biomimesis. To bypass the need to explicitly model diffusion and track each Drug’s height in MediaSpace, values chosen for *pExitMedia* are much smaller than *pExitCell*. To simulate a coarse-grained mechanism for a well-mixed environment, each Drug contained in MediaSpace is reassigned to a random grid point in MediaSpace at the end of each simulation cycle. To simulate the relatively large volume of media compared to cells, each grid point in CellSpace and MediaSpace has a capacity that controls the number of Solutes allowed within. The parameter *scale* controls the capacity of CellSpace: a grid point in CellSpace can contain a number of Solutes equal to the value of *scale*. A grid point in MediaSpace can contain any number of Solutes.

The ISHC takes measurements at the beginning of each simulation cycle, which maps to removing aliquots of media at pre-specified times. Specifically, it reports the combined number of Drug objects contained in CellSpace and MediaSpace. Intrinsic clearance is calculated at the end of each simulation using the equations below, and values are averaged over 16 simulations.

One simulation cycle maps to 1 minute of wet-lab (clock) time. (Contrast this with the ISL time scale, in which 1 simulation cycle maps to 0.25 seconds.) Quantitative similarity criteria were established (see Results) to ensure that in silico results fell within acceptable similarity of wet-lab values. The ISHC achieved validation targets drawn from propranolol intrinsic clearance data from Griffin and Houston and Lavé et al. [33],[52].

### Calculating intrinsic clearance

Intrinsic clearance was calculated assuming mono-exponential depletion of substrate. This assumption is reasonable when drug concentrations are much less than the Michaelis constant, *K*_*m*_. Under this assumption, intrinsic clearance can be calculated in two ways. It can be found using exponential regression fit to the following curve [34]:

(1) $$c\left(t\right)=c\left(0\right)\cdot \exp\left(-\mathrm{CL}\cdot D\cdot t\right),$$

where *c* is the concentration of drug, *CL* is the intrinsic clearance (in μL/min/10^6^ cells), *D* is the cell density (in 10^6^ cells/μL), and *t* is the model time (in min). Alternatively, it can be found using the dose to AUC ratio, where AUC is the area under the concentration-time curve:

(2) $$\mathrm{CL}=\frac{\mathrm{dose}}{\mathrm{AUC}}$$

The dose is equal to the initial concentration normalized by the cell density. Thus, when the AUC is normalized by the initial concentration, the dose to AUC ratio simplifies to:

(3) $$\mathrm{CL}=\frac{1}{D\cdot {\mathrm{AUC}}_{\mathrm{normalized}}}$$

The AUC is theoretically calculated from time zero to infinity or until the concentration reaches zero. Since the validation data take time points until 120 min, the formula for intrinsic clearance can be adjusted using the normalized AUC from zero to 120 min. Assuming a mono-exponential depletion:

(4) $$\mathrm{CL}=\frac{1-f_{120\;\min}}{D\cdot {\mathrm{AUC}}_{\mathrm{normalized},\;0\;\mathrm{to}\;120\;\min}},$$

where *f*_120 min_ is the fraction of drug remaining at 120 min [53].

Both sets of validation data used the dose to AUC ratio to calculate intrinsic clearance. In designing analogs, in silico methods should mimic the wet-lab methods where possible, including measurements and calculation of derived measures. Thus, for these validation data, it is appropriate to determine in silico intrinsic clearance using the dose to AUC ratio. AUC was calculated using the linear trapezoid rule.

### In silico experiments

In silico experiments were run for 200 to 400 simulation cycles, depending on the analog (ISL vs. ISHC) and use case. In silico results reported are averages ± standard deviation of 16 Monte Carlo trials. Results analysis, including averaging among Monte Carlo trials, time-course plots, calculation of intrinsic clearance (ISHC only), and comparison of in silico results to validation data was performed using scripts written in R [54].

### Relational grounding

The units, dimensions, and/or objects to which a variable or model constituent refers establish groundings. During development, grounding decisions impact model flexibility, adaptability, and reusability [55]. Guidelines about groundings are typically built into use case statements and requirements. Because PMMs are designed for straightforward reuse within a broad range of model contexts, it is especially important that grounding decisions in PMMs be carefully considered. Most equation-based models, including pharmacokinetic compartment models, rely upon absolute grounding: model variables, parameters, and input-out are expressed in real-world units. So doing complicates expanding the model to include additional phenomena, changing the model context, integrating multiple models, or reusing model components—all desirable goals of PMMs. In contrast, ISL and ISHC components are grounded relationally: variables, parameters, and input–output are represented in terms of other system components. So doing facilitates exchanging or recombining components and switching model contexts [1],[55]. Relationally grounded models require a separate analog-to-referent mapping protocol. For example, in ISHC experiments here, 1 simulation cycle maps to 1 minute of wet-lab (clock) time. Our requirements include the ability to use simulation measurements of the same analog to achieve validation targets from different wet-lab experiments. So doing requires different analog-to-referent mapping protocols, which is facilitated by employing relational grounding.

## Results and discussion

### Verification of modularization process

Modularization is part of the analog mechanism engineering process, unrelated to referent biological mechanisms; from a software perspective, it is an organizational feature. Thus, the modularization process should not significantly affect analog behavior; it affects the phenomenal analogy but not the structural analogy. To verify that the modularization process did not interfere with simulation results, ISL in silico experiments were run before and after modularization using the same parameters, *including random number seed*. Experiments were run over a wide range of parameterizations. In all cases, the two outputs are identical (see Additional file 2). Though the ISL is largely stochastic, MASON’s random number generator produces duplicable simulations; thus, our modularization implementation resulted in not only statistically indistinguishable but also *identical* simulation results.

### ISL validation experiments

ISL in silico experiments were run for 400 simulation cycles, which maps to 100 seconds of wet-lab time. This time range is consistent with wet-lab measurements of outflow profile for a single-pass, in situ perfused rat liver. The ISL validation target is that at least 80% of outflow values fall within ±1 standard deviation of corresponding wet-lab values. The modularized ISL successfully achieved that validation target for propranolol [30]. A representative outflow profile is provided in Additional file 2. Note that the outputs before and after modularization are identical.

### ISHC validation experiments

ISHC experiments ran for 120 simulation cycles, which maps to 120 minutes of wet-lab time. That duration is similar to those used in vitro to measure intrinsic clearance. There are two quantitative measures of similarity for the ISHC. First, intrinsic clearance must fall within ±1 standard deviation (or ±10%, when no standard deviation is reported) of the wet-lab value. Second, to verify the shape of the concentration-time curve, an acceptable exponential regression fit required an *r*^2^ value of at least 0.95. Wet-lab values for propranolol intrinsic clearance exhibit considerable variability, ranging from 8.9 to 51.0 μL/min/10^6^ cells [33],[52]. The ISHC successfully achieved validation targets for both values, demonstrating its flexibility in covering a large range of measured values.

**Figure 4:**
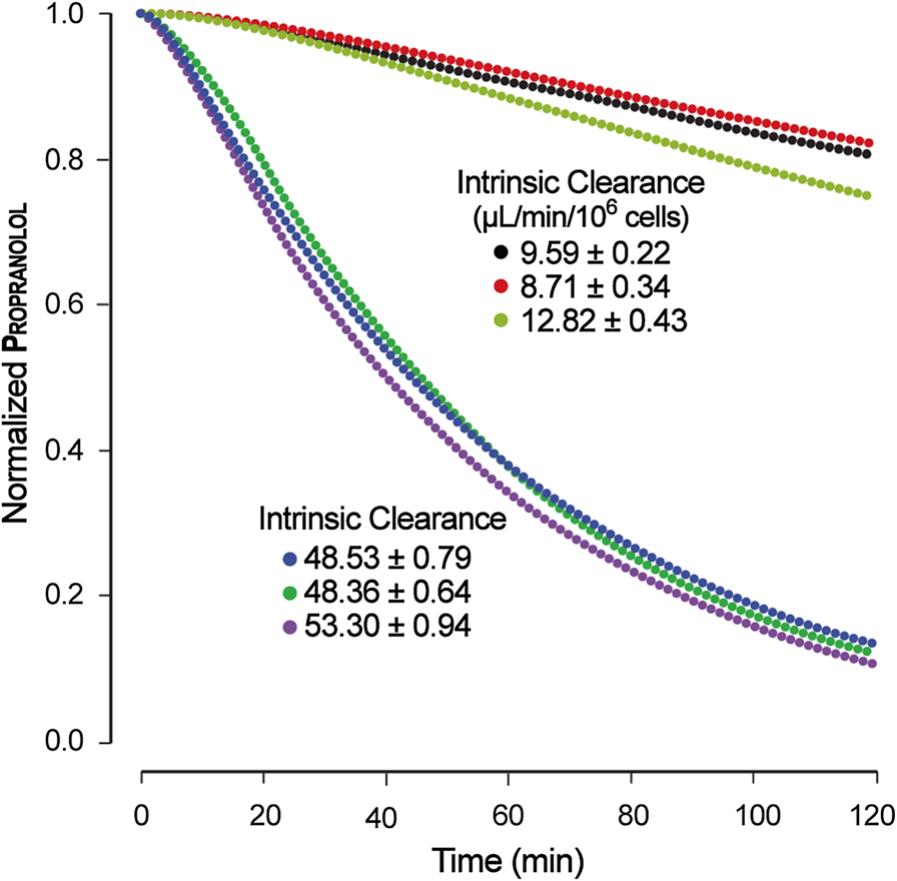
**Propranolol concentration profiles for validating runs of the ISHC.** Points from each curve are averages from 16 Monte Carlo trials. The top three plots achieve validation targets drawn from Griffin and Houston (*CL* = 8.9 ± 4.2 μL/min/10^6^ cells). The bottom three plots achieve validation targets drawn from Lavé et al. (*CL* = 51 μL/min/10^6^ cells).

Various parameterizations were found that satisfy the similarity criteria. Time-course plots of several successfully validating experiments are shown in Figure 4. The relevant parameters among runs and corresponding intrinsic clearance values are shown in Table 3. For all validating runs, *r*^2^ values of the exponential regression fit were greater than 0.98. Although various parameterizations satisfied the similarity criteria, there are differences in the microstructure of the time-course plots. We anticipate that some or all of these parameterized ISHCs will be falsified when additional targeted attributes are included.

**Table 3:** Relevant ISHC parameters and corresponding in silico intrinsic clearance values among validating runs

	**Griffin and Houston[**[33]**]**			**Lavé et al.[**[52]**]**		
	**(*CL*** **= 8.9 ± 4.2 μL/min/10**^**6**^**cells)**			**(*CL*** **= 51.0 μL/min/10**^**6**^**cells)**		
**Experiment number**	1	2	3	4	5	6
***pExitMedia***	0.02	0.02	0.03	0.2	0.18	0.2
***pExitCell***	1.0	1.0	1.0	1.0	1.0	0.85
***pBind***	0.25	0.25	0.25	0.25	0.5	0.5
***pMetabolize***	0.5	0.25	0.25	0.5	0.5	0.5
***dosage***	10000	10000	10000	10000	10000	10000
***CL*** **(μL/min/10**^**6**^**cells)**	9.59 ± 0.22	8.71 ± 0.34	12.82 ± 0.43	48.53 ± 0.79	48.36 ± 0.64	53.30 ± 0.94

One can posit a variety of plausible explanations for the large variability in these and other reported wet-lab intrinsic clearance measurements. For example, differences in hepatocyte heterogeneity among experiments are expected to affect intrinsic clearance measures. In this case, a contributing factor may be the difference in culture geometry. While the calculation for intrinsic clearance is normalized by cell density (cells per volume) and initial substrate concentration, it does not account for the experiment geometry, i.e. the ratio between height of the culture medium (in mm) and area of the well or dish bottom (in cm^2^). The following is a parsimonious theory limited to culture geometry. When this ratio is small (i.e. thin layer of culture medium), drug must travel a relatively short distance to reach cells, resulting in a larger measured value of intrinsic clearance. When this ratio is large, cells are closer together but drug must travel much farther, resulting in a smaller measured value of intrinsic clearance. For Griffin and Houston, who used 400 μL of medium and a 48-well plate, the ratio is approximately 9.64 mm/cm^2^. Lavé et al. used 3 mL of medium but the dimensions of their culture dishes are unspecified. Had they used 6-well or 12-well plates, we would obtain values of 0.33 and 1.99 mm/cm^2^, respectively. Thus, the ratio is likely much smaller for Lavé et al., providing a plausible explanation for the much larger measure of intrinsic clearance. We can make corresponding adjustments to ISHC analogs and mechanisms if and when additional information (like culture geometry) becomes available.

The above explanation is supported by the fact that validating parameterizations for Griffin and Houston required relatively small values for *pExitMedia* (e.g. contrast experiments 1 and 4 in Table 3). Thus, Drugs enter CellSpace much less frequently, lowering the frequency of metabolism events. This information is consistent with the hypothesis that the larger height to area ratio contributes to smaller intrinsic clearance.

### PMMs are robust to changes in model context

PMMs are designed to mimic the underlying referent biological mechanism without being tied to model context. For example, they are not intrinsically tied to a particular time mapping; rather, they are tied to the time mapping of their client model (e.g. ISL, ISHC). Drug clearance experiments span considerably different time scales in vivo (seconds to minutes) versus in vitro (minutes to hours). Thus, there is a corresponding fold difference in the simulation cycle-to-time mapping between the ISL and ISHC. That the pharmacodynamic response module can achieve validation targets for both experimental contexts demonstrates that the modules are robust to changes in analog-to-time mappings.

There is over a five-fold discrepancy in intrinsic clearance values between Griffin and Houston and Lavé et al., though their experimental protocols are very similar. Such experimental variability, coupled with uncertainty arising from other sources, is a troubling reality. Such differences in absolute, quantitative wet-lab measures have real explanations. While we may speculate explanations based on experimental details or in silico results, we typically lack the precise knowledge required to systematically reduce that uncertainty. For M&S methods to be able to achieve validation targets for the same or similar experimental protocols, it is desirable to use models, modules, and methods that enable spanning these ranges of wet-lab measures. That the ISHC can achieve validation targets for both intrinsic clearance values using the same PMMs demonstrates that flexibility.

### Phenotype and mechanism overlap

When object-oriented software engineering methods are used to implement a concrete mechanism, the product of the process is an extant hypothesis: analogs produce a mechanism upon execution. By doing so, we have instantiated (represented with a sequence of concrete instances) a mechanism in silico. A consequence of mechanism execution will be measureable phenomena that are similar (or not) to pre-specified phenomena, such as a response following exposure to a xenobiotic. We measure simulation features; those measurements enable testing the hypothesis. If phenomena meet pre-specified similarity criteria, then the simulation stands as a challengeable, tested theory about mechanistic events at a coarse grain level that may have occurred during the wet-lab experiments [27].

**Figure 5:**
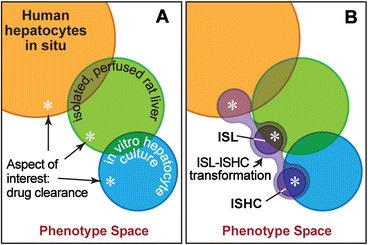
**Phenotype overlap. A)** Shaded circles represent sets of measured values of a subset of all phenotypic attributes. Asterisks represent the conceptual aspect of interest: drug clearance. They encompass the set of derived measures related to drug clearance, though measurements and their generative mechanisms may different among model systems. There is clear overlap of some measured attributes of an isolated, perfused rat liver (green circle) and corresponding human hepatocytes in situ (orange circle). The same can be said of in vitro hepatocyte culture cells (blue circle) and an isolated, perfused rat liver. In non-overlapping regions, the mapping between related attributes is complex. **B)** The ISL and ISHC (dark purple circles) are in silico analogs with their own measurable phenotypes. Overlapping regions represent targeted attributes that have achieved quantitative measures of similarity. The light purple connecting the two analogs illustrates that the transformation between the ISL and ISHC need not be one-to-one. Exploring ISL-ISHC transformations may be instructive of the transformation that occurs between when in vivo cells are isolated into in vitro cultures.

The ISL and ISHC include PMMs that encapsulate individual mechanisms and structural components that simulate objects and space. Executing each analog instantiates the mechanisms, producing measurable phenomena: outflow profile for the ISL and intrinsic clearance for the ISHC. We have demonstrated that the phenomena meet pre-specified similarity criteria based on results from both in vivo and in vitro experiments. The ISL and ISHC share certain phenotypic attributes with their wet-lab counterparts. Their in silico mechanisms thus stand as challengeable, plausible explanations of referent mechanisms of drug response that may have occurred during the wet-lab experiments. These conclusions are explicated below, driven by schematics of overlap in phenotype and mechanism among different models systems (illustrated in Figure 5).

An implicit hypothesis of wet-lab, biomedical experiments, illustrated in Figure 5A, is that attributes of the objects or system of study (e.g. mammalian cells) measured in a particular, controlled experimental context (e.g. in vitro) are quantitatively similar to corresponding attributes in particular humans. However, such precise phenotypic overlap is rare. Attributes of mammalian epithelial cells in one experimental context—a particular wet-lab use case—may be absent in another. A precondition for achieving the long-term goal of simulating human component attributes is having analogs capable of mimicking different wet-lab use cases characterized by different areas of phenotypic overlap. For example, there are similarities in drug response attributes between in vitro and in vivo systems, but there are few with precise, quantitative, one-to-one mappings. We have demonstrated that different regions of phenotypic overlap can be achieved with two analogs of different structure, use case, and quantitative validation targets, but which use the same PMMs and separate simulation-to-validation target mappings.

Though the ISHC contains Hepatocytes that are mechanistically similar to those used by the ISL, they need not map one-to-one with ISL Hepatocytes. This robustness enables a transformation between analogs (light purple in Figure 5B). We hypothesize that ISL-ISHC transformations may be instructive of differences in related attributes between in vitro and in vivo systems. When researchers isolate hepatocytes from livers to study them in culture, in vitro-in vivo extrapolations are embedded in considerable uncertainty. M&S methods can help shrink those uncertainties if validated analog Tissues can be disaggregated into Cells that separately achieve validation targets drawn from in vitro data. We would not assume that attributes are unchanged when the Cells are “reassembled” into Tissue. However, we do know that the tissue cells transformed themselves into what was observed in vitro. In silico, possibly drawing on other wet-lab observations, we can explore plausible explanatory analog transformations. In doing so we can shrink the set of analog options and build confidence in transformation explanations. At a minimum, the validated in vitro parameterizations can serve as a starting point in the search for granularity-specific parameterizations, which achieve new tissue or organ validation targets.

We now move our focus from phenotype space to mechanism space. While the in silico experimental context (i.e. model structure) differed between the ISL and ISHC, the PMMs were identical. Yet, they separately achieved validation targets for different wet-lab systems (in vivo vs. in vitro) with different measures of drug clearance (outflow profile vs. intrinsic clearance). For this to be the case, we assume that some aspects of the generative mechanisms are similar between isolated, perfused rat livers and in vitro hepatocyte culture cells. Taken together, these observations support our claim that pharmacodynamic behaviors of PMMs during execution have similarities with both wet-lab counterparts—that is, they are biomimetic during execution.

### Additional benefits of modularity

We identify six additional benefits of PMMs that are unique or especially important to multi-scale, biomedical M&S:

(1) PMMs enable concrete component-to-biological counterpart mappings. State information is grouped into physiologically meaningful groupings. Modules require (as parameters) these groups according to what has been found to influence the referent mechanism. As a result, non-physiological, mechanism-specific data required for software implementation purposes (e.g. counters or indices) are kept hidden from the modeler. Thus, model components are made explicit, intuitive, and easily understood.

(2) PMMs increase transparency to wet-lab biologists. For wet-lab researchers to easily follow, interpret, and challenge—unassisted—on simulation details, models must be transparent in both representation and execution—form and function. Transparency in representation is achieved by using physiologically meaningful components that map concretely to biological counterparts. Transparency in execution is achieved because interacting model components produce discrete events with hypothesized biological counterparts.

(3) PMMs facilitate separate validation of individual components. Each module can achieve validation targets for data gathered from a variety of wet-lab experimental protocols. Components can be tested in isolation or within the broader model context. When modules are falsified, modularity simplifies the reengineering involved in iterative refinement. As demonstrated here, pharmacodynamic response PMMs can achieve validation targets for data drawn from both in vitro and in vivo drug metabolism experiments. So doing improves face validity of mechanisms and trust in their surviving analogs.

(4) PMMs can be exchanged “on the fly”—that is, during a particular simulation. The PMMs being used by an entity can therefore differ at different simulation cycles. Thus, the set of PMMs being used can be considered part of the entity’s state information. This capability has various applications. Mechanisms can switch among different versions (or be turned “off” or “on”) based on an entity’s state at a given simulation cycle. Different types of state transitions can lead to different versions of mechanisms. For example, a cell analog that transitions from a normal to diseased state during a simulation can switch between corresponding normal or diseased versions of PMMs. Disease progression and spread can be measured during a simulation. Mechanisms can also switch between coarse- and fine-grained versions when specified events occur. Future progeny of current PMMs can exhibit increasingly tunable mechanistic resolution by including additional “nested” PMMs.

(5) PMMs can be exchanged between in silico experiments. In this case it is also useful to think of the set of PMMs being used as part of an analog’s parameterization. Separate in silico experiments can test hypotheses about the differences between normal and diseased states, active and inactive states, coarse- and fine-grained mechanisms, etc.

(6) PMMs enable biomedical domain knowledge to be embedded within concrete analogs. The first type of knowledge embedded in PMMs is the types of physiological information that have been found to directly affect a biological mechanism. This type of knowledge is made explicit because the information is partitioned into physiologically meaningful groupings. It is embedded in logic and algorithms of a PMM, apart from its implementation in a particular analog like the ISL or ISHC. This knowledge will likely be further generalized when additional cell types (e.g. Heart Cell) falsify existing PMMs. In such cases, PMMs may require additional physiological information via new or altered parametric containers. In this way, the falsification of one cell type may inform models of other cell types. The second type of embedded knowledge arises from the implementation of a PMM in a specific model. This knowledge is flexible because each model defines the parametric containers independently, resulting in different mechanisms for different analogs. For example, we embed the knowledge that in vivo hepatocytes experience a gradient in available resources based on their distance from the portal vein, whereas in vitro hepatocytes have uniform resources because they directly contact well-mixed culture media. As new mechanistic insight accumulates, knowledge may be embedded in new or altered versions of PMMs, which can be easily exchanged. Thus, models utilizing PMMs can be concrete instantiations of current understanding about the referent biological system in a particular experimental context.

### Limitations and goals

The modularization methods presented here have several limitations. They are currently limited to object-oriented models, though they could be adapted to functional or logical programming paradigms. Alternative strategies exist for modularizing mathematical models [17],[22]. Including modular mathematical models within or alongside object-oriented ones is feasible but may present technical challenges. Mathematical PMMs therefore require additional M&S methods. The Java-specific implementation we used for the ISL and ISHC is limited to programming environments supporting software interfaces (e.g. Java, C++, C#) or something similar (e.g. Objective-C has protocols, Scala has traits).

A long-term goal of building PMMs is to facilitate development of an analog-based knowledge repository. Such a repository will be an easily accessible, organized framework feature [1]. It will contain annotated records of analogs (current and falsified) and their mechanisms, along with records of in silico experiments. To be both useful and productive, analog components within the repository should be modular. Thus, PMMs are expected to be integral repository components.

Multi-scale models like the ISL and ISHC are designed to have a long lifetime. They are perpetual works in progress whose set of targeted attributes will expand according to iterative model refinement and new validation data. During this process, new PMMs will develop that are increasingly biomimetic during execution and generate phenomena that overlap increasingly large areas in phenotype space. In parallel, we anticipate that PMMs will be adopted, reused, and repurposed by in silico analogs of different cell and tissue types.

## Conclusions

We present a modularization method that defines analog modules based on referent physiological mechanisms. Doing so facilitates separate validation of individual components, enables facile component exchange during or between simulations, and allows analogs to become increasingly transparent, flexible, and biomimetic. The ISHC demonstrates the feasibility of PMMs and their usefulness across multiple model use cases. The pharmacodynamic response module developed here is robust to changes in model context and flexible in its ability to achieve validation targets in the face of considerable experimental uncertainty. Adopting the modularizations methods presented here is expected to facilitate model reuse and integration, thereby accelerating the pace of biomedical research.

## Abbreviations

(M&S): Modeling and simulation

(DEVS): Discrete event systems specification

(PMM): Physiomimetic mechanism module

(ISL): In silico liver

(ISHC): In silico hepatocyte culture

(IR Protocol): Iterative refinement protocol

(*CL*): Intrinsic clearance

(AUC): Area under the concentration-time curve

(SBML): Systems Biology Markup Language

## Competing interests

The authors declare that they have no competing interests.

## Authors’ contributions

BKP modularized the ISL, developed the ISHC, performed the simulations, generated the figures, and drafted the manuscript. GEPR developed the ISL, helped generate the figures, and helped draft the manuscript. CAH directed the study, helped generate the figures, and helped draft the manuscript. All authors read and approved the final manuscript.

## Additional files

## Supplementary Material

Additional file 1: **Sample abridged Java code for each of the three key software aspects: physiomimetic parametric container, PMM, and mechanism user.** The code is intended to illustrate the Java-specific implementation of the three software aspects. For simplicity, it omits many mechanism details, only leaving those features important to the modularization process. Thus, the code is not intended to compile as is. Click here for file

Additional file 2: **Characteristic ISL propranolol outflow profile before and after modularization.** The band represents ±1 standard deviation from the wet-lab validation data. The results before and after modularization are identical; thus, all black data points completely overlap red data points. Click here for file

## References

[B1] Hunt CA Kennedy RC Kim SH Ropella GE Agent-based modeling: a systematic assessment of use cases and requirements for enhancing pharmaceutical research and development productivity Wiley Interdiscip Rev Syst Biol Med 2013 5 461 480 10.1002/wsbm.1222 23737142 PMC3739932

[B2] Hartwell LH Hopfield JJ Leibler S Murray AW From molecular to modular cell biology Nature 1999 402 C47 C52 10.1038/35011540 10591225

[B3] Barba M Dutoit R Legrain C Labedan B Identifying reaction modules in metabolic pathways: bioinformatics deduction and experimental validation of a new putative route in purine catabolism BMC Syst Biol 2013 7 1 99 10.1186/1752-0509-7-99 24093154 PMC4016543

[B4] McCullagh E Farlow J Fuller C Girard J Lipinski-Kruszka J Lu D Noriega T Rollins G Spitzer R Todhunter M Not all quiet on the noise front Nat Chem Biol 2009 5 10 699 704 10.1038/nchembio.222 19763097

[B5] Reynolds JF Acock B Modularity and genericness in plant and ecosystem models Ecol Modell 1997 94 1 7 16 10.1016/S0304-3800(96)01924-2

[B6] Jones J Keating B Porter C Approaches to modular model development Agric Syst 2001 70 2 421 433 10.1016/S0308-521X(01)00054-3

[B7] Iwata K Onosato M Teramoto K Osaki S A modeling and simulation architecture for virtual manufacturing systems CIRP Ann Manuf Technol 1995 44 1 399 402 10.1016/S0007-8506(07)62350-6

[B8] Zeigler BP Praehofer H Kim TG Theory of modeling and simulation: integrating discrete event and continuous complex dynamic systems 2000 Academic Press, San Diego, CA

[B9] Zeigler BP Hierarchical, modular discrete-event modeling in an object-oriented environment Simulation 1987 49 5 219 230 10.1177/003754978704900506

[B10] Pidd M: **Reusing simulation components: simulation software and model reuse: a polemic.** In *Proceedings of the 34*^*th*^*Conference on Winter Simulation: Exploring new Frontiers.* San Diego, CA: Winter Simulation Conference; 2002.

[B11] Kasputis S, Ng HC: **Model composability: formulating a research thrust: composable simulations.** In *Proceedings of the 32*^*nd*^*Conference on Winter Simulation.* Orlando, FL: Society for Computer Simulation International; 2000.

[B12] Robinson S Nance RE Paul RJ Pidd M Taylor SJE Simulation model reuse: definitions, benefits and obstacles Simul Modell Pract Theory 2004 12 7 479 494 10.1016/j.simpat.2003.11.006

[B13] Darden L Thinking again about biological mechanisms Philos Sci 2008 75 5 958 969 10.1086/594538

[B14] Xu C Li CY-T Kong A-NT Induction of phase I, II and III drug metabolism/transport by xenobiotics Arch Pharm Res 2005 28 3 249 268 10.1007/BF02977789 15832810

[B15] Guengerich FP Common and uncommon cytochrome P450 reactions related to metabolism and chemical toxicity Chem Res Toxicol 2001 14 6 611 650 10.1021/tx0002583 11409933

[B16] Blinov ML Ruebenacker O Moraru II Complexity and modularity of intracellular networks: a systematic approach for modelling and simulation IET Syst Biol 2008 2 5 363 368 10.1049/iet-syb:20080092 19045831 PMC2675916

[B17] Mallavarapu A Thomson M Ullian B Gunawardena J Programming with models: modularity and abstraction provide powerful capabilities for systems biology J R Soc Interface 2009 6 32 257 270 10.1098/rsif.2008.0205 18647734 PMC2659579

[B18] Mirschel S Steinmetz K Rempel M Ginkel M ProMot: modular modeling for systems biology Bioinformatics 2009 25 5 687 689 10.1093/bioinformatics/btp029 19147665 PMC2647835

[B19] Chandran D Bergmann FT Sauro HM Computer-aided design of biological circuits using TinkerCell Bioeng Bugs 2010 1 4 274 281 10.4161/bbug.1.4.12506 21327060 PMC3026467

[B20] Snoep JL Bruggeman F Olivier BG Westerhoff HV Towards building the silicon cell: a modular approach Biosystems 2006 83 2 207 216 10.1016/j.biosystems.2005.07.006 16242236

[B21] An G Mi Q Dutta-Moscato J Vodovotz Y Agent-based models in translational systems biology Wiley Interdiscip Rev Syst Biol Med 2009 1 2 159 171 10.1002/wsbm.45 20835989 PMC3640333

[B22] Palsson S Hickling TP Bradshaw-Pierce EL Zager M Jooss K Peter J Spilker ME Palsson BO Vicini P The development of a fully-integrated immune response model (FIRM) simulator of the immune response through integration of multiple subset models BMC Syst Biol 2013 7 1 95 10.1186/1752-0509-7-95 24074340 PMC3853972

[B23] Liu G Qutub AA Vempati P Gabhann FM Popel AS Module-based multiscale simulation of angiogenesis in skeletal muscle Theor Biol Med Model 2011 8 6 1 26 10.1016/j.jtbi.2010.09.039 21463529 PMC3079676

[B24] Sheikh-Bahaei S Hunt CA Enabling clearance predictions to emerge from in silico actions of quasi-autonomous hepatocyte components Drug Metab Dispos 2011 39 10 1910 1920 10.1124/dmd.111.038703 21768275

[B25] Douvin I Zalta EN Abduction The Stanford Encyclopedia of Philosophy ᅟ http://plato.stanford.edu/archives/spr2011/entries/abduction/URL = http://plato.stanford.edu/archives/spr2011/entries/abduction/

[B26] http://plato.stanford.edu/archives/fall2013/entries/reasoning-analogy Bartha P: **Analogy and Analogical Reasoning.** In *The Stanford Encyclopedia of Philosophy*, Fall 2013 Edition. Edited by Zalta EN. URL = .

[B27] Hunt CA Ropella GE Lam TN Tang J Kim SH Engelberg JA Sheikh-Bahaei S At the biological modeling and simulation frontier Pharmacol Res 2009 26 11 2369 2400 10.1007/s11095-009-9958-3 PMC2763179 19756975

[B28] Goresky CA Kinetic interpretation of hepatic multiple-indicator dilution studies Am J Physiol Gastrointest Liver Physiol 1983 245 1 G1 G12 10.1152/ajpgi.1983.245.1.G1 6346902

[B29] Varin F Huet P-M Hepatic microcirculation in the perfused cirrhotic rat liver J Clin Invest 1985 76 5 1904 10.1172/JCI112186 4056057 PMC424238

[B30] Hung DY Chang P Weiss M Roberts MS Structure-hepatic disposition relationships for cationic drugs in isolated perfused rat livers: transmembrane exchange and cytoplasmic binding process J Pharmacol Exp Ther 2001 297 2 780 789 11303070

[B31] Goresky CA A linear method for determining liver sinusoidal and extravascular volumes Am J Physiol 1963 204 4 626 640 13949263 10.1152/ajplegacy.1963.204.4.626

[B32] Chou C Evans AM Fornasini G Rowland M Relationship between lipophilicity and hepatic dispersion and distribution for a homologous series of barbiturates in the isolated perfused in situ rat liver Drug Metab Dispos 1993 21 5 933 938 7902258

[B33] Griffin SJ Houston JB Prediction of in vitro intrinsic clearance from hepatocytes: comparison of suspensions and monolayer cultures Drug Metab Dispos 2005 33 1 115 120 15608354 10.1124/dmd.33.1.

[B34] Naritomi Y Terashita S Kagayama A Sugiyama Y Utility of hepatocytes in predicting drug metabolism: comparison of hepatic intrinsic clearance in rats and humans in vivo and in vitro Drug Metab Dispos 2003 31 5 580 588 10.1124/dmd.31.5.580 12695346

[B35] Brian Houston J Utility of in vitro drug metabolism data in predicting in vivo metabolic clearance Biochem Pharmacol 1994 47 9 1469 1479 10.1016/0006-2952(94)90520-7 8185657

[B36] Rane A Wilkinson G Shand D Prediction of hepatic extraction ratio from in vitro measurement of intrinsic clearance J Pharmacol Exp Ther 1977 200 2 420 424 839445

[B37] Park S Kim SH Ropella GE Roberts MS Hunt CA Tracing multiscale mechanisms of drug disposition in normal and diseased livers J Pharmacol Exp Ther 2010 334 1 124 136 10.1124/jpet.110.168526 20406856

[B38] Zhu BT On the general mechanism of selective induction of cytochrome P450 enzymes by chemicals: some theoretical considerations Expert Opin Drug Metab Toxicol 2010 6 4 483 494 10.1517/17425250903578642 20113197 PMC2842473

[B39] Winther RG: **The map analogy.** *Trans Inst Brit Geogr* 2014, in press.

[B40] Yilmaz L Ören TI Discrete-event multimodels and their agent-supported activation and update Proceedings of the Agent-Directed Simulation Symposium of the Spring Simulation Multiconference (SMC’05) 2005

[B41] Bierman G Hicks M Sewell P Stoyle G Formalizing dynamic software updating Proceedings of the Second International Workshop on Unanticipated Software Evolution (USE) 2003

[B42] Ropella GE Kennedy RC Hunt CA Falsifying an enzyme induction mechanism within a validated, multiscale liver model Int J Agent Technol Syst 2012 4 3 1 14 10.4018/jats.2012070101

[B43] Jungermann K Kietzmann T Oxygen: modulator of metabolic zonation and disease of the liver Hepatology 2000 31 2 255 260 10.1002/hep.510310201 10655244

[B44] Beier K Völkl A Metzger C Mayer D Bannasch P Fahimi H Hepatic zonation of the induction of cytochrome P450 IVA, peroxisomal lipid beta-oxidation enzymes and peroxisome proliferation in rats treated with dehydroepiandrosterone (DHEA). Evidence of distinct zonal and sex-specific differences Carcinogenesis 1997 18 8 1491 1498 10.1093/carcin/18.8.1491 9276621

[B45] Allen JW Bhatia SN Formation of steady-state oxygen gradients in vitro: Application to liver zonation Biotechnol Bioeng 2003 82 3 253 262 10.1002/bit.10569 12599251

[B46] Luke S Cioffi-Revilla C Panait L Sullivan K Mason: A new multi-agent simulation toolkit Proceedings of the 2004 SwarmFest Workshop 2004

[B47] Kim SH Park S Ropella GE Hunt CA Agent-based simulation of drug disposition in cirrhotic liver Proceedings of the 2010 Spring Simulation Multiconference 2010 Society for Computer Simulation International, Orlando, FL

[B48] Lam TN Hunt CA Discovering plausible mechanistic details of hepatic drug interactions Drug Metab Dispos 2009 37 1 237 246 10.1124/dmd.108.023820 18936110

[B49] Yan L Ropella GE Park S Roberts MS Hunt CA Modeling and simulation of hepatic drug disposition using a physiologically based, multi-agent in silico liver Pharmacol Res 2008 25 5 1023 1036 10.1007/s11095-007-9494-y 18044012

[B50] Yan L Sheihk-Bahaei S Park S Ropella GE Hunt CA Predictions of hepatic disposition properties using a mechanistically realistic, physiologically based model Drug Metab Dispos 2008 36 4 759 768 10.1124/dmd.107.019067 18227144

[B51] Mizuno N Niwa T Yotsumoto Y Sugiyama Y Impact of drug transporter studies on drug discovery and development Pharmacol Rev 2003 55 3 425 461 10.1124/pr.55.3.1 12869659

[B52] Lavé T Dupin S Schmitt C Chou R Jaeck D Coassolo P Integration of in vitro data into allometric scaling to predict hepatic metabolic clearance in man: application to 10 extensively metabolized drugs J Pharmacol Sci 1997 86 5 584 590 10.1021/js960440h 9145383

[B53] Lau YY Sapidou E Cui X White RE Cheng K-C Development of a novel in vitro model to predict hepatic clearance using fresh, cryopreserved, and sandwich-cultured hepatocytes Drug Metab Dispos 2002 30 12 1446 1454 10.1124/dmd.30.12.1446 12433818

[B54] R: A language and environment for statistical computing R Foundation for Statistical Computing 2012

[B55] Hunt CA Ropella GE ning Lam T Gewitz AD Relational grounding facilitates development of scientifically useful multiscale models Theor Biol Med Model 2011 8 35 1 22 21951817 10.1186/1742-4682-8-35 PMC3200146

