# Supplementary figures and images for: Toward modular biological models: defining analog modules based on referent physiological mechanisms
Source: BMC Syst Biol. 2014 Aug 16;8:95. doi: 10.1186/s12918-014-0095-1 (PMC4236728; doi:10.1186/s12918-014-0095-1)

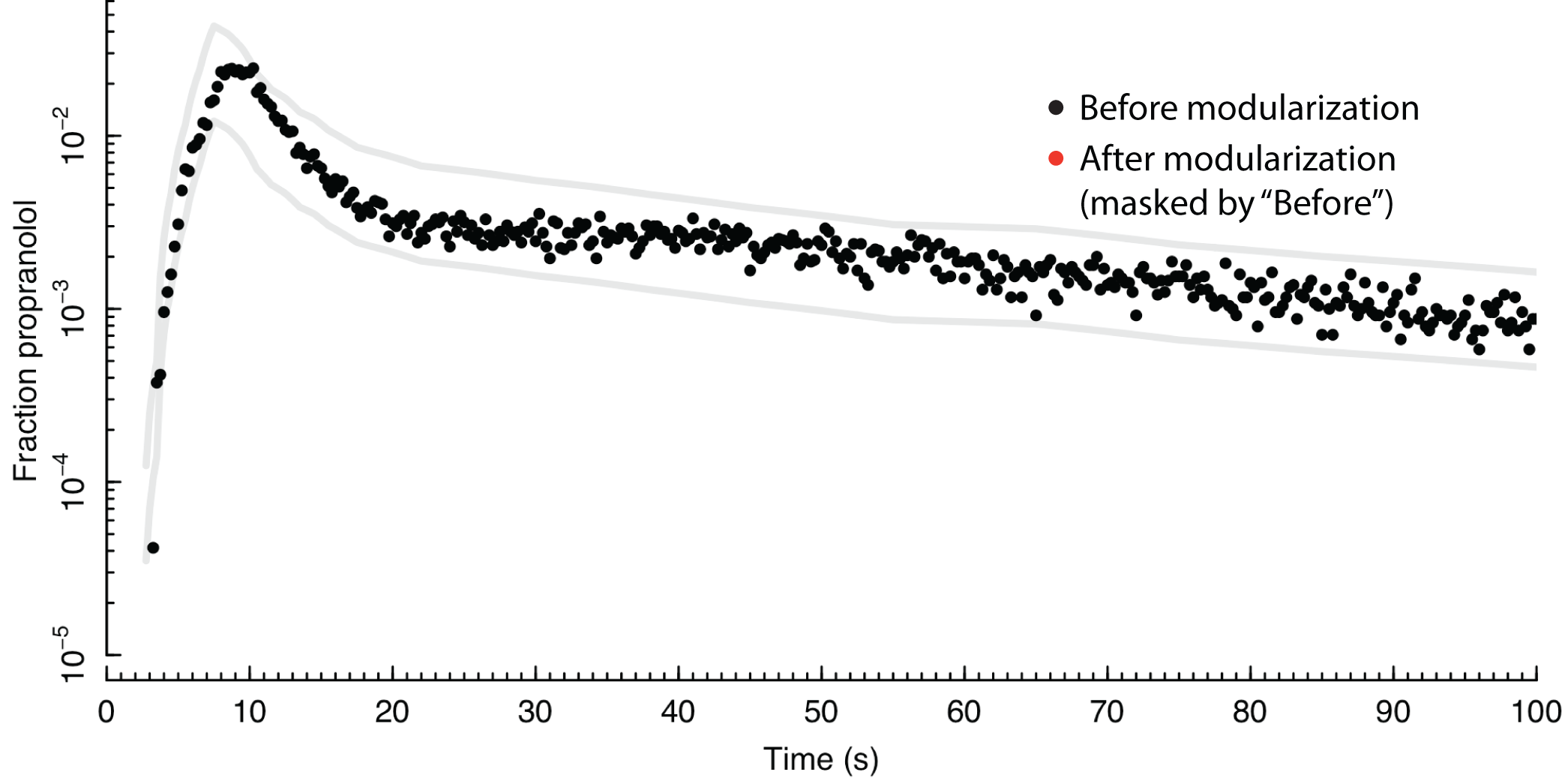

Supplement: Additional file 2: — Characteristic ISL propranolol outflow profile before and after modularization. The band represents ±1 standard deviation from the wet-lab validation data. The results before and after modularization are identical; thus, all black data points completely overlap red data points. [file s12918-014-0095-1-S2.tiff]
